# Risks Posed by Reston, the Forgotten Ebolavirus

**DOI:** 10.1128/mSphere.00322-16

**Published:** 2016-12-28

**Authors:** Diego Cantoni, Arran Hamlet, Martin Michaelis, Mark N. Wass, Jeremy S. Rossman

**Affiliations:** a School of Biosciences, University of Kent, Canterbury, United Kingdom; b Department of Infectious Disease Epidemiology, MRC Centre for Outbreak Analysis and Modelling, Imperial College London, London, United Kingdom; University of Pittsburgh School of Medicine

**Keywords:** Ebolavirus, pathogenicity, Reston

## Abstract

Out of the five members of the *Ebolavirus* family, four cause life-threatening disease, whereas the fifth, Reston virus (RESTV), is nonpathogenic in humans. The reasons for this discrepancy remain unclear. In this review, we analyze the currently available information to provide a state-of-the-art summary of the factors that determine the human pathogenicity of Ebolaviruses. RESTV causes sporadic infections in cynomolgus monkeys and is found in domestic pigs throughout the Philippines and China. Phylogenetic analyses revealed that RESTV is most closely related to the Sudan virus, which causes a high mortality rate in humans. Amino acid sequence differences between RESTV and the other Ebolaviruses are found in all nine Ebolavirus proteins, though no one residue appears sufficient to confer pathogenicity. Changes in the glycoprotein contribute to differences in Ebolavirus pathogenicity but are not sufficient to confer pathogenicity on their own. Similarly, differences in VP24 and VP35 affect viral immune evasion and are associated with changes in human pathogenicity. A recent *in silico* analysis systematically determined the functional consequences of sequence variations between RESTV and human-pathogenic Ebolaviruses. Multiple positions in VP24 were differently conserved between RESTV and the other Ebolaviruses and may alter human pathogenicity. In conclusion, the factors that determine the pathogenicity of Ebolaviruses in humans remain insufficiently understood. An improved understanding of these pathogenicity-determining factors is of crucial importance for disease prevention and for the early detection of emergent and potentially human-pathogenic RESTVs.

## Abstract (precis)

Out of the five members of the *Ebolavirus* family, four cause life-threatening disease, whereas the fifth, Reston virus (RESTV), is nonpathogenic in humans.

## INTRODUCTION

The recent Ebola virus (EBOV) outbreak in West Africa changed our perception of the global threat posed by the Ebolaviruses. The outbreak was of unprecedented size, resulting in 28,657 confirmed cases and 11,325 deaths (as of 5 August 2016 [http://www.who.int]), with several reported deaths on other continents (1). Previous Ebolavirus outbreaks ranged from a very few infected individuals to a few hundred cases (2). During this outbreak, evidence has emerged that EBOVs were able to persist and remain infective in immune-privileged sites in the body (including the eye, semen, vaginal fluid, and breast milk) for over 6 months after disease resolution and clearance of the virus from the bloodstream, significantly complicating disease containment and control (3, 4). The combination of these factors (outbreak size and virus persistence) raises significant concern for the danger posed by future outbreaks. Advancing our understanding of Ebolaviruses is extremely important in order to ensure adequate surveillance and outbreak containment; however, much remains unknown about the mechanisms by which these viruses cause disease.

Ebolaviruses are filoviruses (filamentous viruses) with a single-stranded negative-sense RNA genome. The *Ebolavirus* family consists of five species, *Zaire ebolavirus* (type virus, EBOV), *Sudan ebolavirus* (type virus, Sudan virus [SUDV]), *Tai Forest ebolavirus* (type virus, Tai Forest virus [TAFV]), and *Bundibugyo ebolavirus* (type virus, Bundibugyo virus [BDBV]), and *Reston ebolavirus* (type virus, Reston virus, RESTV). EBOV, SUDV, TAFV, and BDBV cause severe hemorrhagic disease in humans, with mortality rates ranging from 50 to 90% (5, 6). RESTV is mildly virulent in pigs, avirulent in humans, but lethal in nonhuman primates (NHPs), although African green monkeys (*Chlorocebus aethiops*) are resistant to RESTV infection and baboons (*Papio hamadryas*) are resistant to both RESTV and EBOV infections (7–11). Coinfection with other pathogenic viruses may also have a role in the modulationof RESTV disease severity, as simian hemorrhagic fever virus has been found in fatal cases of RESTV infections in NHPs, though the contribution of each pathogen to the overall disease remains unknown (12).

The Ebolavirus genome is approximately 19 kb in length and encodes nine proteins, nucleoprotein (NP), glycoprotein (GP), soluble GP (sGP), small soluble GP (ssGP), RNA-dependent RNA polymerase (L), and structural proteins VP24, VP30, VP35, and VP40, many of which are associated with viral pathogenicity (Table 1) (13–15). Each of the viral proteins shows a high degree of sequence conservation among the different Ebolavirus species, and no single protein appears to be sufficient to confer a pathogenic phenotype on RESTV. As a result, the risks of RESTV mutating into a human-pathogenic strain remain unknown and therefore, the virus remains classified as a biosafety level 4 pathogen.

**TABLE:** Protein components of Ebolavirus^a^

Protein	Function	% of RESTV residues identified as SDPs
NP	Protects and packages the viral genome by encapsidation	3.87
GP	Class I viral fusion protein, responsible for binding and entry into host cells, activated by proteolysis, creating GP1 and GP2; GP1,2 has extensive roles in modulation of the immune response and alteration of the expression of cell surface adhesion molecules; cleavage of GP1,2 from the plasma membrane creates a soluble variant	4.3
sGP	Possible roles in immune evasion and alteration of endothelial permeability	2.43
ssGP	Unknown	Not determined
VP24	Secondary matrix protein, minor component of virions; key player in pathogenicity, inhibits components of immune response	3.59
VP30	Viral nucleocapsid component; key role in transcription depending on its state of phosphorylation	5.86
VP35	Polymerase cofactor in transcription and replication; prevents antiviral response in cells by blocking IRF-3 and protein kinase EIF2AK2/PKR	5.57
VP40	Regulates viral transcription, morphogenesis, packaging, and budding	2.72
Polymerase	Replicates the viral genome	2.95

a The percentage of SDP sites in RESTV, compared to EBOV, may offer clues to the lack of RESTV pathogenicity in humans, though higher levels of SDPs do not necessarily indicate a change in protein function or activity. Furthermore, the percentage of difference is likely to fluctuate regularly because of viral mutation and evolution (49, 58, 65–69).

**FIG 1:**
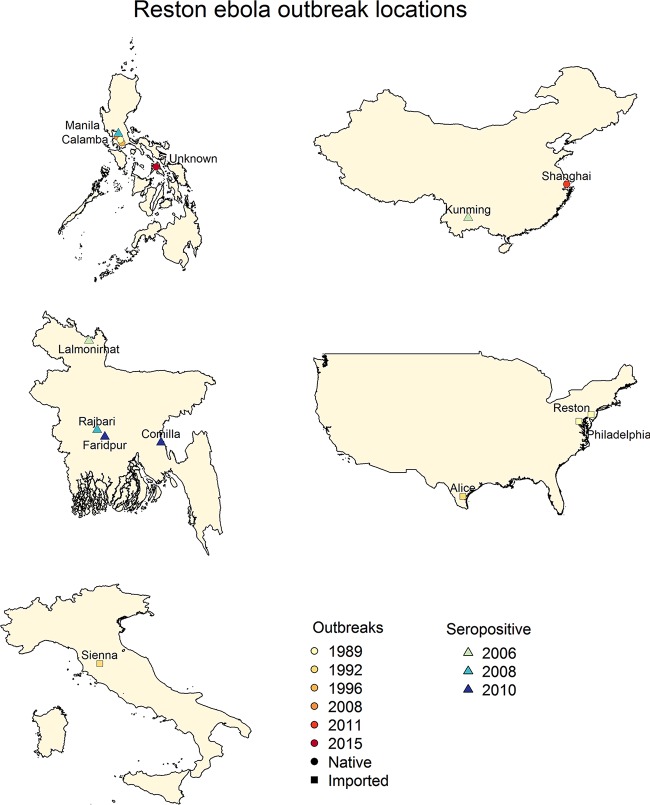
Detection of RESTV. The maps shown indicate the locations of RESTV detection, either viral RNA or seropositive evidence, that suggest that RESTV is more widely distributed than previously thought (7, 18, 19, 25). The distribution of RESTV appears to be in close proximity to the equator, similar to that of other Ebolaviruses, although RESTV has never been detected in Africa.

While the four human-pathogenic *Ebolavirus* species are all found in Africa, RESTV is known to be endemic to the Philippines and China. This makes RESTV the only Ebolavirus known to exist outside Africa to date. RESTV was discovered by electron microscopic examination of infected cells during the 1989 epizootic outbreak in cynomolgus monkeys that had been imported from the Philippines into the United States and housed at a research facility in Reston, VA (16). The monkeys displayed the hallmark symptoms of Ebolavirus disease, including subcutaneous hemorrhaging, bloody diarrhea, and sudden onset of anorexia (12). In contrast, four handlers in the United States who became infected with RESTV did not show any signs or symptoms of illness, nor did the seropositive handlers at the Laguna export facility in the Philippines (17). Since then, several known minor outbreaks of RESTV have occurred in monkeys (Fig. 1): a subsequent outbreak in 1990 in Reston, VA, in which four handlers developed antibodies to RESTV; a 1992 outbreak in Sienna, Italy, in monkeys imported from the same facility in the Philippines that caused the 1989 outbreak; a 1996 outbreak in Alice, TX, at the Texas Primate Center; and two outbreaks in 1996 and 2015 in the Philippines (12, 16).

In 2008, RESTV was found in farmed pigs in Manila, the Philippines (8) (Table 2). Six handling personnel were found to be seropositive for RESTV, suggesting RESTV transmission from pigs to humans. Interestingly, RESTV was only found in sick pigs that were also infected with porcine reproductive and respiratory syndrome virus (PRRSV), although histological analysis did not reveal colocalization of the two viruses at any body site. Whether RESTV contributed to the manifested symptoms remains to be determined (9). The viral genome sequences isolated from pigs in 2008 exhibited a 2.5% mean difference in nucleotide sequence identity from the 1989 Reston monkey isolate. Three RESTV samples recently taken from infected pigs at different geographical locations in the Philippines (Panganisan and Bulacan) showed even greater divergence from each other, with a 3.93% mean difference in nucleotide sequence identity (8). It was suggested that the reason for this genetic diversity could be that both monkeys and pigs were infected from different unidentified reservoirs (8). In 2012, RESTV was again detected in pigs with PRRSV, this time in China, with 96.1 to 98.9% sequence similarity to previous pig and monkey isolates from the Philippines (18).

**TABLE 2:** Outbreaks of *Reston ebolavirus*^a^

Location	Yr	Organism	No. of seropositive humans
RESTV outbreaks			
Philippines	1989–1990	Cynomolgus monkey	3
United States (VA, PA)	1989–1990	Cynomolgus monkey	0
United States (TX)	1989–1990	Cynomolgus monkey	4
Italy	1992–1993	Cynomolgus monkey	0
United States (TX)	1996	Cynomolgus monkey	0
Philippines	1996	Cynomolgus monkey	1
Philippines	2008	Pig	6
China	2011	Pig	0
Philippines	2015	Cynomolgus monkey	0
Locations with seropositive evidence only			
Philippines	2008–2009	Fruit bat	
China	2006–2009	Fruit bat	
Bangladesh	2010–2011	Fruit bat	

a The 1989 outbreak was characterized by high mortality rates in cynomolgus monkeys, whereas infected pigs were found to be coinfected with PRRSV. No human handlers were reported to show any symptoms of disease (7, 8, 17, 19, 70, 71).

Despite the fact that the first known RESTV outbreak occurred almost 30 years ago, there is still relatively little known about this virus. This includes the natural reservoirs of RESTV, the route of transmission from this reservoir to pigs and monkeys, and the reasons underlying its lack of pathogenicity in humans. Because of its similarity to the other four Ebolaviruses, there is a concern that RESTV could mutate to become pathogenic in humans and that this Ebolavirus could then spread easily around the world through imported livestock or other animal hosts. In this review, we will discuss potential reservoirs for RESTV, its genetic relationship to other Ebolaviruses, and the molecular basis for its lack of pathogenicity in humans. We will also speculate on the potential risk of RESTV to human health and how this can be addressed.

## RESTV HOSTS AND RESERVOIRS

Circulation of RESTV in reservoir species and other hosts may increase the probability that human-pathogenic RESTV variants will emerge, in particular if selective pressures exerted by different hosts cause viral mutation or if the host range results in more frequent contact with humans. To date, it is known that RESTV can infect humans, NHPs, and pigs. However, it is often suggested that there are reservoirs of this virus that have not yet been identified (8, 19). Bats are the most commonly implicated reservoirs of filoviruses (20–22). In 2008 and 2009, *Rousettus amplexicaudatus* fruit bats possessing RESTV-specific IgG antibodies were captured in the Philippine forests of Diliman and Cuezon, located within 60 km of the Bulacan farm where RESTV-infected monkeys were identified in 2008 (23). *R. amplexicaudatus* bats are genetically similar to *R. aegyptiacus* bats, which are thought to be the reservoir of Ebolaviruses in Africa (24). In addition, RESTV, as well as EBOV, antibodies have been found in Bangladesh and China in the related bat species *R. leschenaultia*, suggesting that the Ebolaviruses may circulate in a wide geographical area (19, 25). While live virus has not been detected in these bats, it is possible that the bats could rapidly clear the viral infections or restrict viral replication to levels that are below the limit of detection.

RESTV was also detected in domestic pigs in Shanghai, China, that were coinfected with PRRSV. The RESTV sequence was found to be 96.1 to 98.9% identical to that of strains previously found in domestic pigs and monkeys in the Philippines (18). Experimental infections with RESTV, in the absence of PRRSV, have also been performed to examine the disease course in pigs. Interestingly, infected pigs were found to have high viral loads in the lungs and were able to shed the virus through the nasopharynx, though the pigs showed no disease symptoms (9). This further demonstrates that pigs are a hosts of RESTV and suggests that, at least in pigs, RESTV may be able to spread through aerosol transmission. Thus, continued RESTV spread to humans through contact with domestic animals may increase the likelihood of RESTV adaption and the possible emergence of a human-pathogenic, aerosol-transmittable RESTV.

## RESTV GENOME EVOLUTION

**FIG 2:**
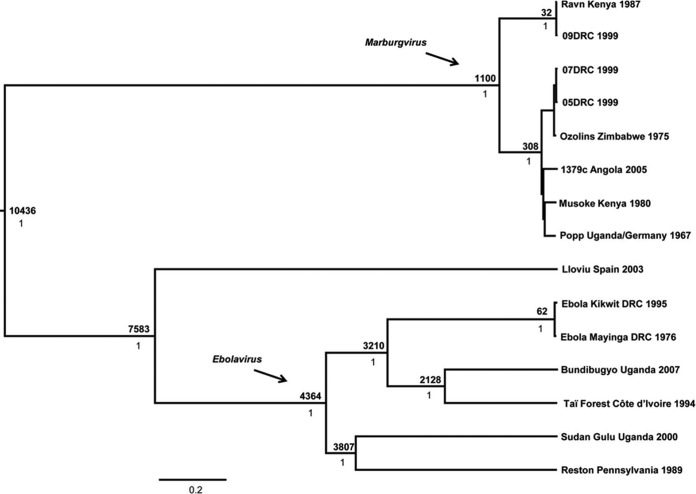
Phylogenetic analysis of the *Filoviridae* family. Shown are the results of a Bayesian coalescent analysis of viruses in the *Filoviridae* family showing that RESTV is most closely related to SUDV (27).

RESTV is thought to have originated in Africa and to have diverged from SUDV about 1,400 to 1,600 years ago before it migrated toward Asia (Fig. 2) (6, 26, 27). The hypothesis that filoviruses have spread beyond the African continent was recently reinforced by the discovery of a new filovirus in bats in the Lloviu caves of Spain, as well as the presence of RESTV in bats, pigs, and NHPs in Asia (18, 22, 25).

A recent phylogenetic study analyzed seven RESTV genomes, including four that were obtained from infected pigs (27). While the virus showed a genetic change of 0.079% in 1 year on the same farm, there was a divergence of up to 4.5% on a different farm (27). This study also showed that RESTV evolves at a rate of 8.21 × 10^−4^ nucleotide substitutions/site/year, similar to that of EBOV and much higher than the rate of nucleotide substitutions of SUDV, which could make the virus more susceptible to adaptation to humans.

The overall selection pressures between EBOV and RESTV show that amino acids on the main viral antigenic determinant, GP, were under increased selective pressure. EBOV selection pressure was found to be 0.299, whereas RESTV showed 0.329, whereby a ratio of >1 indicates increased selection and <1 indicates decreased selection (28). The EBOV GP showed selective pressure at mucin domain residues 377 and 443, whereas RESTV GP was only under selective pressure at one glycosylated residue in the GP1 glycan cap, N229, though this residue was under stronger selection that any in EBOV GP (29). These changes in GP may result in a different host tropism or may affect immune evasion, which may be a cause for concern about RESTV, though this has not been experimentally demonstrated.

## DIFFERENCES THAT MAY CONTRIBUTE TO PATHOGENICITY

A number of studies have compared human-pathogenic Ebolaviruses to RESTV in order to identify the underlying reasons for the observed differences in human pathogenicity (30–32). One of the proteins implicated in pathogenesis, VP24, acts by antagonizing the host innate immune response. VP24 binds to karyopherin 1 (KPNA1), KPNA5, and KPNA6, inhibiting the nuclear import of phosphorylated (active) STAT1 and restricting the expression of interferon-stimulated genes (ISGs) (33). VP24 was also found to reduce the binding of heterogeneous nuclear ribonuclear protein complex C1/C2 (hnRNP C1/C2) to KPNA1, further restricting phosphorylated-STAT1 nuclear import, as well as relocating hnRNP C1/C2 from the nucleus into the cytoplasm (34). In viruses such as poliovirus and human papillomavirus, this relocation facilitates viral RNA replication and the translation of viral proteins (34–36). In addition to blocking the STAT1 pathway, VP24 may also directly bind to STAT1 to prevent its nuclear import (37). EBOV VP24 may be more effective at suppressing the host interferon response than RESTV VP24, as EBOV-infected cells express lower levels of many ISGs than do RESTV-infected cells (38).

Several VP24 amino acid differences between EBOV and RESTV may affect the virus’s ability to inhibit STAT1 signaling, thus affecting pathogenicity (39). These variant residues appear to cluster at key sites involved in VP24 binding to KPNAs, such as the VP24 142-to-146 loop. In this region, RESTV displays conserved amino acid changes (M136L, Q139R, R140S) compared to other Ebolavirus species (30). Changing the RESTV S140 residue to R140 modifies the hydrophobic moment of the protein and appears to be sufficient to enable KPNA binding (32). These findings suggest that specific changes in RESTV VP24 may affect interactions with KPNAs, resulting in a reduced ability to inhibit interferon signaling. In 6- to 8-week-old STAT1 knockout BALB/c mice, both EBOV and RESTV infections resulted in disease manifestation, causing lethargy, weight loss, and decreased survival rates after 6 days postinfection. However, wild-type BALB/c mice (6 to 8 weeks old) showed no manifestation of disease upon infection with either EBOV or RESTV (40, 41). EBOV was found to be lethal only in newborn mice or following several rounds of adaptation; however, comparable experiments have not been performed with RESTV and thus the ability of RESTV to adapt and cause disease in mice remains unknown (40, 41). In contrast to the STAT1^−/−^ infections, RESTV infection of alpha/beta interferon receptor (IFNAR) knockout mice resulted in only transient weight loss, whereas EBOV infection was uniformly lethal (42). Interestingly, following symptom resolution, RESTV-infected IFNAR^−/−^ mice showed protection against a subsequent challenge with mouse-adapted EBOV (43). These results demonstrate the complexity of investigating Ebolavirus infections and RESTV pathogenicity in mice.

Bioinformatic investigation determined amino acid residues that are differently conserved (specificity-determining positions [SDPs]) between RESTV and the four human-pathogenic *Ebolavirus* species (44, 45). Several of these SDPs were located on protein surfaces, suggesting their possible involvement in molecular interactions (46). While the VP24 sequence identity between EBOV and RESTV is 80%, only 9 of 251 residues were identified as SDPs, possibly contributing to RESTV’s lack of pathogenicity in humans (15). Of the nine SDPs found in VP24, three (T131S, M136L, and Q139R) are located at the KPNA5 binding site. This supports the hypothesis that RESTV VP24 may be less effective at karyopherin binding and suppressing the interferon response. In addition, another SDP in RESTV VP24 results in the loss of hydrogen bonding between T226 and D48, potentially impacting protein stability and function (46). However, the SDPs were not restricted to VP24 and many SDPs were found in other protein interfaces that may affect interactions and stability (Table 1).

VP35 is an interferon antagonist that inhibits the activation of interferon regulatory factor 3 (IRF3) following the sensing of viral RNA by the pattern recognition receptor RIG-I. RESTV VP35 has 65% sequence identity with EBOV VP35 and shows 19 SDPs (46–49). Although, it was found that both RESTV and EBOV VP35 molecules were able to inhibit IRF-3 activation, blocking the IRF3-dependent transcription of ISGs 54 and 56. In addition, neither RESTV nor EBOV VP35 could block signaling from the IFNAR (50). This implies that not all SDPs have an effect on pathogenicity; therefore, the consequences of these differences are not clear.

In addition to VP24, differences in the GP may also affect viral pathogenesis (51, 52). EBOV GP contains a mucin-like domain that increases blood vesicle permeability by downregulating the expression of integrin β1 and other cell adhesion molecules (53–55). RESTV GP has several conserved SDPs (R325G, H354L, Q403P, S418E, T448P) and was found to have a significantly weaker influence in downregulating integrin β1 expression, compared to EBOV GP (46, 55). When it was examined *in vivo*, it was seen that the presence of the RESTV GP attenuated EBOV pathogenicity, whereas the reverse genetic conversion of RESTV GP to EBOV GP was not sufficient to confer a pathogenic phenotype on RESTV, indicating that other proteins are involved in the regulation of Ebolavirus pathogenicity (38, 42, 55).

The functions of the two soluble and secreted Ebolavirus proteins sGP and ssGP remain the most elusive, with the structure of EBOV sGP only recently being solved (56). sGP shares 295 N-terminal residues with GP; thus, sGP is thought to contribute to evasion of the humoral system by absorbing GP antibodies (57, 58). In addition, sGP seems to play an anti-inflammatory role by promoting recovery of the endothelial barrier during Ebolavirus infection (59). RESTV appears to secrete more sGP than EBOV, suggesting that the anti-inflammatory role of sGP has a more significant role in pathogenicity, considering its role in restoration of the endothelial barrier (59). At 37 kDa, RESTV ssGP is significantly larger than that of the other Ebolaviruses (33 kDa). However, the potential involvement of ssGP in pathogenicity remains unclear and thus the effect of the RESTV ssGP extension is unknown (60).

It may also be that lack of RESTV virulence in humans is due to a delay in viral transcription and genome replication, as RESTV was found to have slower growth kinetics, suggesting a growth impairment that was not observed with EBOV (61). The organization of the RESTV genome differs from that of the other Ebolaviruses. Ebolaviruses contain gene overlaps between GP and VP30. In contrast, these two genes are separated by an intergenic region in RESTV (26). This change in genomic organization may affect the transcription of GP and VP30 or alter the efficiency of genome replication. Though the relationship between EBOV gene overlap and genomic replication has not been tested, it is possible that the reduced efficiency of RESTV replication, combined with functional protein differences, could enable RESTV to infect humans without causing any detectable pathogenicity.

## CONCLUSIONS

RESTV is unique among the Ebolaviruses in that it does not cause disease in humans. However, RESTV is infectious in several animal species that exist in close contact with humans, and humans can be asymptomatically infected with the virus, raising the question of whether humans can be carriers of Ebolaviruses and suggesting that further adaptation of RESTV could cause a significant risk to human health.

An observed significant factor in the outbreak in West Africa was that infected bush meat provided a route of transmission of virus to humans (62). Humans and *R. leschenaultia* bats in Bangladesh share a food source, date palm sap, which may be a potential route of viral transmission to humans. In addition, the ability of pigs to become hosts of RESTV means that the virus can be established in the human food chain, which is a cause for concern, as prolonged human contact may play a role in virus adaptation to humans.

Furthermore, it may be the case that single amino acid substitutions in SDP sites can affect pathogenicity. This is concerning, as many RESTV proteins had only a few SDPs that differed from those of EBOV, suggesting that a minimal number of mutations may be required to restore RESTV pathogenicity in humans. Thus, the investigation of the effects of individual SDPs is of great importance for understanding EBOV pathogenicity.

While the likelihood that RESTV will become pathogenic in humans is not clear, given that it can establish itself in the human food chain in densely populated areas, the potential risk that the virus poses to human health worldwide is significant. This risk is even greater when considering that because RESTV is nonpathogenic in humans, the only people who have been screened for RESTV infection have worked at monkey and pig farms undergoing RESTV outbreaks; thus, the actual prevalence of RESTV in human and animal populations may be significantly greater than anticipated. However, in response to the recent outbreak of EBOV in West Africa, research into Ebolavirus therapeutics has shown promising advances, in particular, vaccines and an antibody for pan-Ebolavirus therapy that is able to protect mice from lethal EBOV infections (63, 64) and may be able to prevent and mitigate future outbreaks of any Ebolavirus species, including RESTV.

## References

[1] CDC. 2016 2014 Ebola outbreak in West Africa—case counts. Centers for Disease Control and Prevention, Atlanta, GA http://www.cdc.gov/vhf/ebola/outbreaks/2014-west-africa/case-counts.html Accessed 20 April 2016.

[2] Georges AJ, Leroy EM, Renaut AA, Benissan CT, Nabias RJ, Ngoc MT, Obiang PI, Lepage JP, Bertherat EJ, Bénoni DD, Wickings EJ, Amblard JP, Lansoud-Soukate JM, Milleliri JM, Baize S, Georges-Courbot MC 1999 Ebola hemorrhagic fever outbreaks in Gabon, 1994–1997: epidemiologic and health control issues. J Infect Dis 179(Suppl 1):S65–S75. doi:10.1086/514290. 9988167

[3] Deen GF, Knust B, Broutet N, Sesay FR, Formenty P, Ross C, Thorson AE, Massaquoi TA, Marrinan JE, Ervin E, Jambai A, McDonald SLR, Bernstein K, Wurie AH, Dumbuya MS, Abad N, Idriss B, Wi T, Bennett SD, Davies T, Ebrahim FK, Meites E, Naidoo D, Smith S, Banerjee A, Erickson BR, Brault A, Durski KN, Winter J, Sealy T, Nichol ST, Lamunu M, Ströher U, Morgan O, Sahr F 14 10 2015 Ebola RNA persistence in semen of Ebola virus disease survivors—preliminary report. N Engl J Med doi:10.1056/NEJMoa1511410. PMC5798881 26465681

[4] Varkey JB, Shantha JG, Crozier I, Kraft CS, Lyon GM, Mehta AK, Kumar G, Smith JR, Kainulainen MH, Whitmer S, Ströher U, Uyeki TM, Ribner BS, Yeh S 2015 Persistence of Ebola virus in ocular fluid during convalescence. N Engl J Med 372:2423–2427. doi:10.1056/NEJMoa1500306. 25950269 PMC4547451

[5] Kuhn JH, Becker S, Ebihara H, Geisbert TW, Johnson KM, Kawaoka Y, Lipkin WI, Negredo AI, Netesov SV, Nichol ST, Palacios G, Peters CJ, Tenorio A, Volchkov VE, Jahrling PB 2010 Proposal for a revised taxonomy of the family Filoviridae: classification, names of taxa and viruses, and virus abbreviations. Arch Virol 155:2083–2103. doi:10.1007/s00705-010-0814-x. 21046175 PMC3074192

[6] Geisbert TW, Hensley LE 2004 Ebola virus: new insights into disease aetiopathology and possible therapeutic interventions. Expert Rev Mol Med 6:1–24. doi:10.1017/S1462399404008300. 15383160

[7] Rollin PE, Williams RJ, Bressler DS, Pearson S, Cottingham M, Pucak G, Sanchez A, Trappier SG, Peters RL, Greer PW, Zaki S, Demarcus T, Hendricks K, Kelley M, Simpson D, Geisbert TW, Jahrling PB, Peters CJ, Ksiazek TG 1999 Ebola (subtype Reston) virus among quarantined nonhuman primates recently imported from the Philippines to the United States. J Infect Dis 179(Suppl 1):S108–S114. doi:10.1086/514303. 9988173

[8] Barrette RW, Metwally SA, Rowland JM, Xu L, Zaki SR, Nichol ST, Rollin PE, Towner JS, Shieh WJ, Batten B, Sealy TK, Carrillo C, Moran KE, Bracht AJ, Mayr GA, Sirios-Cruz M, Catbagan DP, Lautner EA, Ksiazek TG, White WR, McIntosh MT 2009 Discovery of swine as a host for the Reston ebolavirus. Science 325:204–206. doi:10.1126/science.1172705. 19590002

[9] Marsh GA, Haining J, Robinson R, Foord A, Yamada M, Barr JA, Payne J, White J, Yu M, Bingham J, Rollin PE, Nichol ST, Wang LF, Middleton D 2011 Ebola Reston virus infection of pigs: clinical significance and transmission potential. J Infect Dis 204(Suppl 3):S804–S809. doi:10.1093/infdis/jir300. 21987755

[10] Bente D, Gren J, Strong JE, Feldmann H 2009 Disease modeling for Ebola and Marburg viruses. Dis Model Mech 2:12–17. doi:10.1242/dmm.000471. 19132113 PMC2615158

[11] Fisher-Hoch SP, Lynnette Brammer TL, Trappier SG, Hutwagner LC, Farrar BB, Ruo SL, Brown BG, Hermann LM, Perez-Oronoz GI, Goldsmith CS, Hanes MA, McCormick JB 1992 Pathogenic potential of filoviruses: role of geographic origin of primate host and virus strain. J Infect Dis 166:753–763. doi:10.1093/infdis/166.4.753. 1527410

[12] Dalgard DW, Hardy RJ, Pearson SL, Pucak GJ, Quander RV, Zack PM, Peters CJ, Jahrling PB 1992 Combined simian hemorrhagic fever and Ebola virus infection in cynomolgus monkeys. Lab Anim Sci 42:152–157. 1318446

[13] Feldmann H, Geisbert TW 2011 Ebola haemorrhagic fever. Lancet 377:849–862. doi:10.1016/S0140-6736(10)60667-8. 21084112 PMC3406178

[14] de La Vega MA, Wong G, Kobinger GP, Qiu X 2015 The multiple roles of sGP in Ebola pathogenesis. Viral Immunol 28:3–9. doi:10.1089/vim.2014.0068. 25354393 PMC4287119

[15] Ikegami T, Calaor AB, Miranda ME, Niikura M, Saijo M, Kurane I, Yoshikawa Y, Morikawa S 2001 Genome structure of Ebola virus subtype Reston: differences among Ebola subtypes. Arch Virol 146:2021–2027. doi:10.1007/s007050170049. 11722021

[16] Geisbert TW, Jahrling PB 1990 Use of immunoelectron microscopy to show Ebola virus during the 1989 United States epizootic. J Clin Pathol 43:813–816. doi:10.1136/jcp.43.10.813. 2229429 PMC502829

[17] Centers for Disease Control (CDC) 1990 Update: filovirus infection in animal handlers. MMWR Morb Mortal Wkly Rep 39:221. 2107388

[18] Pan Y, Zhang W, Cui L, Hua X, Wang M, Zeng Q 2014 Reston virus in domestic pigs in China. Arch Virol 159:1129–1132. doi:10.1007/s00705-012-1477-6. 22996641

[19] Yuan J, Zhang Y, Li J, Zhang Y, Wang LF, Shi Z 2012 Serological evidence of ebolavirus infection in bats, China. Virol J 9:236. doi:10.1186/1743-422X-9-236. 23062147 PMC3492202

[20] Leroy EM, Kumulungui B, Pourrut X, Rouquet P, Hassanin A, Yaba P, Délicat A, Paweska JT, Gonzalez JP, Swanepoel R 2005 Fruit bats as reservoirs of Ebola virus. Nature 438:575–576. doi:10.1038/438575a. 16319873

[21] Towner JS, Pourrut X, Albariño CG, Nkogue CN, Bird BH, Grard G, Ksiazek TG, Gonzalez JP, Nichol ST, Leroy EM 2007 Marburg virus infection detected in a common African bat. PLoS One 2:e764. doi:10.1371/journal.pone.0000764. 17712412 PMC1942080

[22] Negredo A, Palacios G, Vázquez-Morón S, González F, Dopazo H, Molero F, Juste J, Quetglas J, Savji N, de la Cruz Martínez M, Herrera JE, Pizarro M, Hutchison SK, Echevarría JE, Lipkin WI, Tenorio A 2011 Discovery of an ebolavirus-like filovirus in Europe. PLoS Pathog 7:e1002304. doi:10.1371/journal.ppat.1002304. 22039362 PMC3197594

[23] Taniguchi S, Watanabe S, Masangkay JS, Omatsu T, Ikegami T, Alviola P, Ueda N, Iha K, Fujii H, Ishii Y, Mizutani T, Fukushi S, Saijo M, Kurane I, Kyuwa S, Akashi H, Yoshikawa Y, Morikawa S 2011 Reston ebolavirus antibodies in bats, the Philippines. Emerg Infect Dis 17:1559–1560. doi:10.3201/eid1708.101693. 21801651 PMC3381561

[24] Taniguchi S, Watanabe S, Masangkay JS, Omatsu T, Ikegami T, Alviola P, Ueda N, Iha K, Fujii H, Ishii Y, Mizutani T, Fukushi S, Saijo M, Kurane I, Kyuwa S, Akashi H, Yoshikawa Y, Morikawa S 2011 Reston Ebolavirus antibodies in bats, the Philippines. Emerg Infect Dis 17:1559–1560. doi:10.3201/eid1708.101693. 21801651 PMC3381561

[25] Olival KJ, Islam A, Yu M, Anthony SJ, Epstein JH, Khan SA, Khan SU, Crameri G, Wang LF, Lipkin WI, Luby SP, Daszak P 2013 Ebola virus antibodies in fruit bats, Bangladesh. Emerg Infect Dis 19:270–273. doi:10.3201/eid1902.120524. 23343532 PMC3559038

[26] Sanchez A 2003 Ebola viruses. *In* eLS John Wiley & Sons Ltd, Chichester, United Kingdom. doi:10.1038/npg.els.0001019.

[27] Carroll SA, Towner JS, Sealy TK, McMullan LK, Khristova ML, Burt FJ, Swanepoel R, Rollin PE, Nichol ST 2013 Molecular evolution of viruses of the family Filoviridae based on 97 whole-genome sequences. J Virol 87:2608–2616. doi:10.1128/JVI.03118-12. 23255795 PMC3571414

[28] Hurst LD 2002 The Ka/Ks ratio: diagnosing the form of sequence evolution. Trends Genet 18:486. doi:10.1016/S0168-9525(02)02722-1. 12175810

[29] Li YH, Chen SP 2014 Evolutionary history of Ebola virus. Epidemiol Infect 142:1138–1145. doi:10.1017/S0950268813002215. 24040779 PMC9151191

[30] Zhang APP, Abelson DM, Bornholdt ZA, Liu T, Woods VL, Saphire EO 2012 The ebolavirus VP24 interferon antagonist: know your enemy. Virulence 3:440–445. doi:10.4161/viru.21302. 23076242 PMC3485981

[31] Reid SP, Valmas C, Martinez O, Sanchez FM, Basler CF 2007 Ebola virus VP24 proteins inhibit the interaction of NPI-1 subfamily karyopherin alpha proteins with activated STAT1. J Virol 81:13469–13477. doi:10.1128/JVI.01097-07. 17928350 PMC2168840

[32] Chakraborty S, Rao BJ, Asgeirsson B, Dandekar AM 2014 Correlating the ability of VP24 protein from Ebola and Marburg viruses to bind human karyopherin to their immune suppression mechanism and pathogenicity using computational methods. F1000Res 3:265. doi:10.12688/f1000research.5666.1.

[33] Reid SP, Leung LW, Hartman AL, Martinez O, Shaw ML, Carbonnelle C, Volchkov VE, Nichol ST, Basler CF 2006 Ebola virus VP24 binds karyopherin alpha1 and blocks STAT1 nuclear accumulation. J Virol 80:5156–5167. doi:10.1128/JVI.02349-05. 16698996 PMC1472181

[34] Shabman RS, Gulcicek EE, Stone KL, Basler CF 2011 The Ebola virus VP24 protein prevents hnRNP C1/C2 binding to karyopherin α1 and partially alters its nuclear import. J Infect Dis 204(Suppl 3):S904–S910. doi:10.1093/infdis/jir323. 21987768 PMC3189985

[35] Brunner JE, Nguyen JHC, Roehl HH, Ho TV, Swiderek KM, Semler BL 2005 Functional interaction of heterogeneous nuclear ribonucleoprotein C with poliovirus RNA synthesis initiation complexes. J Virol 79:3254–3266. doi:10.1128/JVI.79.6.3254-3266.2005. 15731220 PMC1075716

[36] Gontarek RR, Gutshall LL, Herold KM, Tsai J, Sathe GM, Mao J, Prescott C, Del Vecchio AM 1999 hnRNP C and polypyrimidine tract-binding protein specifically interact with the pyrimidine-rich region within the 3′NTR of the HCV RNA genome. Nucleic Acids Res 27:1457–1463. doi:10.1093/nar/27.6.1457. 10037806 PMC148338

[37] Zhang APP, Bornholdt ZA, Liu T, Abelson DM, Lee DE, Li S, Woods VL, Saphire EO 2012 The Ebola virus interferon antagonist VP24 directly binds STAT1 and has a novel, pyramidal fold. PLoS Pathog 8:e1002550. doi:10.1371/journal.ppat.1002550. 22383882 PMC3285596

[38] Kash JC, Mühlberger E, Carter V, Grosch M, Perwitasari O, Proll SC, Thomas MJ, Weber F, Klenk HD, Katze MG 2006 Global suppression of the host antiviral response by Ebola- and Marburgviruses: increased antagonism of the type I interferon response is associated with enhanced virulence. J Virol 80:3009–3020. doi:10.1128/JVI.80.6.3009-3020.2006. 16501110 PMC1395418

[39] Groseth A, Ströher U, Theriault S, Feldmann H 2002 Molecular characterization of an isolate from the 1989/90 epizootic of Ebola virus Reston among macaques imported into the United States. Virus Res 87:155–163. doi:10.1016/S0168-1702(02)00087-4. 12191779

[40] de Wit E, Munster VJ, Metwally SA, Feldmann H 2011 Assessment of rodents as animal models for Reston ebolavirus. J Infect Dis 204(Suppl 3):S968–S972. doi:10.1093/infdis/jir330. 21987777 PMC3189989

[41] Raymond J, Bradfute S, Bray M 2011 Filovirus infection of STAT-1 knockout mice. J Infect Dis 204:S986–S990. doi:10.1093/infdis/jir335. 21987780

[42] Groseth A, Marzi A, Hoenen T, Herwig A, Gardner D, Becker S, Ebihara H, Feldmann H 2012 The Ebola virus glycoprotein contributes to but is not sufficient for virulence in vivo. PLoS Pathog 8:e1002847. doi:10.1371/journal.ppat.1002847. 22876185 PMC3410889

[43] Brannan JM, Froude JW, Prugar LI, Bakken RR, Zak SE, Daye SP, Wilhelmsen CE, Dye JM 2015 Interferon alpha/beta receptor-deficient mice as a model for Ebola virus disease. J Infect Dis 212(Suppl 2):S282–S294. doi:10.1093/infdis/jiv215. 25943199

[44] Casari G, Sander C, Valencia A 1995 A method to predict functional residues in proteins. Nat Struct Biol 2:171–178. doi:10.1038/nsb0295-171. 7749921

[45] Rausell A, Juan D, Pazos F, Valencia A 2010 Protein interactions and ligand binding: from protein subfamilies to functional specificity. Proc Natl Acad Sci U S A 107:1995–2000. doi:10.1073/pnas.0908044107. 20133844 PMC2808218

[46] Pappalardo M, Juliá M, Howard MJ, Rossman JS, Michaelis M, Wass MN 2016 Conserved differences in protein sequence determine the human pathogenicity of Ebolaviruses. Sci Rep 6:23743. doi:10.1038/srep23743. 27009368 PMC4806318

[47] Basler CF, Wang X, Mühlberger E, Volchkov V, Paragas J, Klenk HD, García-Sastre A, Palese P 2000 The Ebola virus VP35 protein functions as a type I IFN antagonist. Proc Natl Acad Sci U S A 97:12289–12294. doi:10.1073/pnas.220398297. 11027311 PMC17334

[48] Cárdenas WB, Loo YM, Gale M, Hartman AL, Kimberlin CR, Martínez-Sobrido L, Saphire EO, Basler CF 2006 Ebola virus VP35 protein binds double-stranded RNA and inhibits alpha/beta interferon production induced by RIG-I signaling. J Virol 80:5168–5178. doi:10.1128/JVI.02199-05. 16698997 PMC1472134

[49] Prins KC, Cárdenas WB, Basler CF 2009 Ebola virus protein VP35 impairs the function of interferon regulatory factor-activating kinases IKKepsilon and TBK-1. J Virol 83:3069–3077. doi:10.1128/JVI.01875-08. 19153231 PMC2655579

[50] Basler CF, Mikulasova A, Martinez-Sobrido L, Paragas J, Mühlberger E, Bray M, Klenk HD, Palese P, García-Sastre A 2003 The Ebola virus VP35 protein inhibits activation of interferon regulatory factor 3. J Virol 77:7945–7956. doi:10.1128/JVI.77.14.7945-7956.2003. 12829834 PMC161945

[51] Feldmann H, Kiley MP 1999 Classification, structure, and replication of filoviruses. Curr Opin Microbiol Immunol 235:1–21. doi:10.1007/978-3-642-59949-1_1. 9893375

[52] Richman DD, Cleveland PH, McCormick JB, Johnson KM 1983 Antigenic analysis of strains of Ebola virus: identification of two Ebola virus serotypes. J Infect Dis 147:268–271. doi:10.1093/infdis/147.2.268. 6827143

[53] Chan SY, Ma MC, Goldsmith MA 2000 Differential induction of cellular detachment by envelope glycoproteins of Marburg and Ebola (Zaire) viruses. J Gen Virol 81:2155–2159. doi:10.1099/0022-1317-81-9-2155. 10950971

[54] Yang ZY, Duckers HJ, Sullivan NJ, Sanchez A, Nabel EG, Nabel GJ 2000 Identification of the Ebola virus glycoprotein as the main viral determinant of vascular cell cytotoxicity and injury. Nat Med 6:886–889. doi:10.1038/78645. 10932225

[55] Simmons G, Wool-Lewis RJ, Baribaud F, Netter RC, Bates P 2002 Ebola virus glycoproteins induce global surface protein down-modulation and loss of cell adherence. J Virol 76:2518–2528. doi:10.1128/jvi.76.5.2518-2528.2002. 11836430 PMC153797

[56] Pallesen J, Murin CD, de Val N, Cottrell CA, Hastie KM, Turner HL, Fusco ML, Flyak AI, Zeitlin L, Crowe JE, Andersen KG, Saphire EO, Ward AB 2016 Structures of Ebola virus GP and sGP in complex with therapeutic antibodies. Nat Microbiol 1:16128. doi:10.1038/nmicrobiol.2016.128. 27562261 PMC5003320

[57] Falzarano D, Krokhin O, Wahl-Jensen V, Seebach J, Wolf K, Schnittler HJ, Feldmann H 2006 Structure-function analysis of the soluble glycoprotein, sGP, of Ebola virus. ChemBioChem 7:1605–1611. doi:10.1002/cbic.200600223. 16977667

[58] Sanchez A, Trappier SG, Mahy BW, Peters CJ, Nichol ST 1996 The virion glycoproteins of Ebola viruses are encoded in two reading frames and are expressed through transcriptional editing. Proc Natl Acad Sci U S A 93:3602–3607. doi:10.1073/pnas.93.8.3602. 8622982 PMC39657

[59] Wahl-Jensen VM, Afanasieva TA, Seebach J, Ströher U, Feldmann H, Schnittler HJ 2005 Effects of Ebola virus glycoproteins on endothelial cell activation and barrier function. J Virol 79:10442–10450. doi:10.1128/JVI.79.16.10442-10450.2005. 16051836 PMC1182673

[60] Mahale KN, Patole MS 2015 The crux and crust of ebolavirus: analysis of genome sequences and glycoprotein gene. Biochem Biophys Res Commun 463:756–761. doi:10.1016/j.bbrc.2015.06.008. 26051281

[61] Boehmann Y, Enterlein S, Randolf A, Mühlberger E 2005 A reconstituted replication and transcription system for Ebola virus Reston and comparison with Ebola virus Zaire. Virology 332:406–417. doi:10.1016/j.virol.2004.11.018. 15661171

[62] Alexander KA, Sanderson CE, Marathe M, Lewis BL, Rivers CM, Shaman J, Drake JM, Lofgren E, Dato VM, Eisenberg MC, Eubank S 2015 What factors might have led to the emergence of Ebola in West Africa? PLoS Negl Trop Dis 9:e0003652. doi:10.1371/journal.pntd.0003652. 26042592 PMC4456362

[63] Holtsberg FW, Shulenin S, Vu H, Howell KA, Patel SJ, Gunn B, Karim M, Lai JR, Frei JC, Nyakatura EK, Zeitlin L, Douglas R, Fusco ML, Froude JW, Saphire EO, Herbert AS, Wirchnianski AS, Lear-Rooney CM, Alter G, Dye JM, Glass PJ, Warfield KL, Aman MJ 2015 Pan-ebolavirus and pan-filovirus mouse monoclonal antibodies: protection against Ebola and Sudan viruses. J Virol 90:266–278. doi:10.1128/JVI.02171-15. 26468533 PMC4702560

[64] Furuyama W, Marzi A, Nanbo A, Haddock E, Maruyama J, Miyamoto H, Igarashi M, Yoshida R, Noyori O, Feldmann H, Takada A 2016 Discovery of an antibody for pan-ebolavirus therapy. Sci Rep 6:20514. doi:10.1038/srep20514. 26861827 PMC4748290

[65] Watanabe S, Noda T, Kawaoka Y 2006 Functional mapping of the nucleoprotein of Ebola virus. J Virol 80:3743–3751. doi:10.1128/JVI.80.8.3743-3751.2006. 16571791 PMC1440433

[66] Lee JE, Saphire EO 2009 Ebolavirus glycoprotein structure and mechanism of entry. Future Virol 4:621–635. doi:10.2217/fvl.09.56. 20198110 PMC2829775

[67] Han Z, Boshra H, Sunyer JO, Zwiers SH, Paragas J, Harty RN 2003 Biochemical and functional characterization of the Ebola virus VP24 protein: implications for a role in virus assembly and budding. J Virol 77:1793–1800. doi:10.1128/JVI.77.3.1793-1800.2003. 12525613 PMC140957

[68] Silva LP, Vanzile M, Bavari S, Aman JMJ, Schriemer DC 2012 Assembly of Ebola virus matrix protein VP40 is regulated by latch-like properties of N and C terminal tails. PLoS One 7:e39978. doi:10.1371/journal.pone.0039978. 22792204 PMC3390324

[69] Li J, Rahmeh A, Morelli M, Whelan SP 2008 A conserved motif in region v of the large polymerase proteins of nonsegmented negative-sense RNA viruses that is essential for mRNA capping. J Virol 82:775–784. doi:10.1128/JVI.02107-07. 18003731 PMC2224588

[70] Jahrling PB, Geisbert TW, Johnson ED, Peters CJ, Dalgard DW, Hall WC, Peters CJ 1990 Preliminary report: isolation of Ebola virus from monkeys imported to USA. Lancet 335:502–505. doi:10.1016/0140-6736(90)90737-P. 1968529

[71] Hayes CG, Burans JP, Ksiazek TG, Del Rosario RA, Miranda ME, Manaloto CR, Barrientos AB, Robles CG, Dayrit MM, Peters CJ 1992 Outbreak of fatal illness among captive macaques in the Philippines caused by an Ebola-related filovirus. Am J Trop Med Hyg 46:664–671. 1621890 10.4269/ajtmh.1992.46.664

